# Oral-Parenteral Conversion Factor for Morphine in Palliative Cancer Care: A Prospective Randomized Crossover Pilot Study

**DOI:** 10.1155/2011/504034

**Published:** 2011-02-15

**Authors:** Jan Starlander, Christina Melin-Johansson, Håkan Jonsson, Bertil Axelsson

**Affiliations:** ^1^Department of Internal Medicine, Östersund Hospital, 831 83 Östersund, Sweden; ^2^Department of Health Science, Mid Sweden University, 831 25 Östersund, Sweden; ^3^Regional Centre of Oncology, Umeå University Hospital, 901 70 Umeå, Sweden; ^4^Department of General Surgery, Östersund Hospital, 831 83 Östersund, Sweden; ^5^The Research and Development Unit, Jämtland County Council, 831 82 Östersund, Sweden; ^6^Department of Radiation Sciences, Umeå University, 901 85 Umeå, Sweden

## Abstract

*Objective*. This pilot study clinically tests whether a conversion factor of 2 to 1 is appropriate when changing from oral to parenteral morphine administration in the treatment of cancer-related nociceptive pain and calculates the size of an adequately powered future study. *Methods*. Eleven outpatients with incurable cancer and well-controlled nociceptive pain were randomly assigned to either intravenous or subcutaneous morphine using half the previous oral 24-hour dose. Each group crossed over after the first three-day period. Serum concentrations of morphine and its metabolites were monitored as well as intensity of pain. *Results*. Oral to subcutaneous and oral to intravenous quotas of morphine concentrations were approximately 0.9. Subcutaneous to intravenous morphine quotas were 1. *Conclusions*. The conversion factor of 2 to 1 seems to be a reasonable average but with an obvious need for individual adjustments. Concurrent medications and substantially higher doses of morphine could potentially affect the appropriate conversion factor. An adequately powered study to validate these findings would need at least 121 patients.

## 1. Background

In palliative cancer care, morphine is still the strong opioid of choice according to recommendations by WHO [1] and EAPC [2], and it is recommended that the morphine be given orally for as long as possible. Difficulties in swallowing due to neurological causes or general weakness during the last days of life are common reasons for changing the administration from oral to parenteral. Occasionally, mechanical problems in the gastrointestinal tract force us to abandon the oral route of administration early in the disease trajectory. When expected survival is reasonably long, a switch to transdermal fentanyl is the least invasive alternative [3]. When death is imminent, subcutaneous morphine administration is preferred by many as it provides more flexibility and a lower possibility of needing to adjust the dose [4]. The clinical task in these situations is to translate the oral morphine dose to a parenteral dose in the safest possible way, that is, to maintain pain control without side effects.

The recommendation used to be a conversion factor from oral to parenteral morphine of 3 to 1 [5], which has also been proposed earlier this year by the Cleveland group [6]. Recently, a conversion factor closer to 2 to 1 has been recommended [7]. There has also been some discussion as to whether subcutaneous and intravenous administration needs different dose calculations. These varying recommendations have created a feeling of uncertainty for prescribing physicians. Our clinical impression is that the factor 2 to 1 works well when changing from stable oral morphine dose to subcutaneous infusion. Complications such as episodes of breakthrough pain and/or signs of overdosing are rare. 

This study investigates the use of a conversion factor of 2 to1 in clinical practice, to evaluate pain control and side effects systematically and to monitor effects on plasma concentrations of morphine, morphine-3-glucoronide (M3G), and morphine-6-glucoronide (M6G). A secondary aim was to examine whether any differences could be detected in plasma concentrations of morphine and its metabolites comparing subcutaneous and intravenous infusion. The hypothesis was that quotas between morphine plasma concentrations after oral administration and subcutaneous or intravenous administration would be approximately 1. A further aim was to use the results of this study to calculate the needed number of patients in a future adequately powered study.

## 2. Methods

### 2.1. Patients

Eleven incurable cancer patients treated at home by the palliative care team in Östersund, Sweden were approached. All were receiving ongoing controlled release morphine therapy (Dolcontin, Pfizer), and their cancer-related pain was well controlled (Numerical Rating Scale (NRS) < 4). We deliberately looked for patients who had a survival prognosis of more than one month and whose cognition was intact. All patients received information about the study and gave informed consent in writing. Ethical approval was given by the Ethical Committee of Umeå University.

### 2.2. Procedure

Blood samples were taken within one hour before the regular morning dose of oral morphine. Plasma concentrations of morphine, M3G, and M6G were analyzed by high-performance liquid chromatography (HPLC) at Huddinge University Hospital. Blood for analysis of creatinine and liver tests were performed.

New plasma concentrations were taken four to five hours after the regular morning dose of oral-controlled release morphine. Patients were then randomly assigned to groups and given either subcutaneous or intravenous continuous infusion of morphine. We used a seven-day INFUSOR produced by Baxter, which delivered 0.5 ml/hour of a mixture equaling half the previous oral dose per 24 hours. Three days later, the groups switched to the other way of parenteral administration using the same 24-hour morphine dose. Plasma concentrations were taken before the switch and again after at least 48 hours of the new mode of administration. Demographical and clinical data were recorded in a study protocol. The Eastern Cooperative Oncology Group Scale of Performance Status (ECOG PS) was used to assess patients' functional status [8]. Body Mass Index (BMI) was calculated, and Numerical Rating Scale (NRS) measurements of pain, nausea, and tiredness were undertaken daily throughout the weeklong study period. An increase in NRS score of >1 was regarded as clinically significant.

### 2.3. Statistics

Statistical comparisons of NRS ratings taken during the different ways of administration were performed using nonparametric the Wilcoxon signed rank test. Mann-Whitney *U* analysis was used when comparing metabolite concentrations between those who experienced more pain during parenteral administration with those without increased pain ratings. 

Power calculations were performed to see how many patients that would be needed to conclude that a conversion factor of 2 is appropriate. An equivalence test was designed in which equivalence of the conversion factor was defined as an interval 2+/−d. Thus, the null hypothesis (nonequivalence) was rejected if there were evidence that the true conversion factor was inside this interval. The log-transformed quota between oral and intravenous concentrations was assumed to be normally distributed with the estimated standard deviation (SD) as a known parameter.

## 3. Results

No patient withdrew from the study during the seven-day study period. The study group of 11 patients consisted of five men and six women. Median age was 71 years (range 58–80). Diagnoses were lung cancer (*n* = 3), prostate cancer (*n* = 3), gynecological cancer (*n* = 2), GI cancer (*n* = 2), and breast cancer (*n* = 1). Median oral morphine dose was 40 mg/24 h (10–200 mg/24 h). Median survival after study completion was 80 days (Interquartile Range, IQR = 138). 

We divided the oral five-hour morphine plasma concentration with the intravenous or the subcutaneous morphine concentration of the same patient forming two quotas.

**Figure 1:**
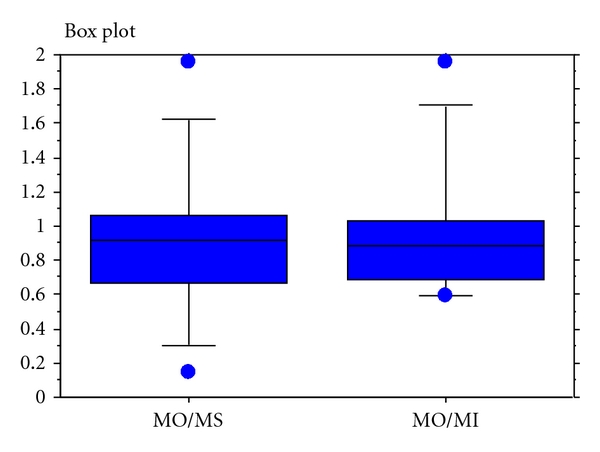
Quotas of oral morphine concentration and subcutaneous morphine: MO/MS, oral morphine concentration and intravenous morphine: MO/MI (*n* = 11).

The median oral to subcutaneous quota for plasma morphine was 0.91 (IQR = 0.39). The median oral to intravenous quota was 0.88 (IQR = 0.39), and the subcutaneous to intravenous quota was 1.0 (IQR = 0.29). The distributions of quotas for the eleven study patients are shown in Figure 1.

**Figure 2:**
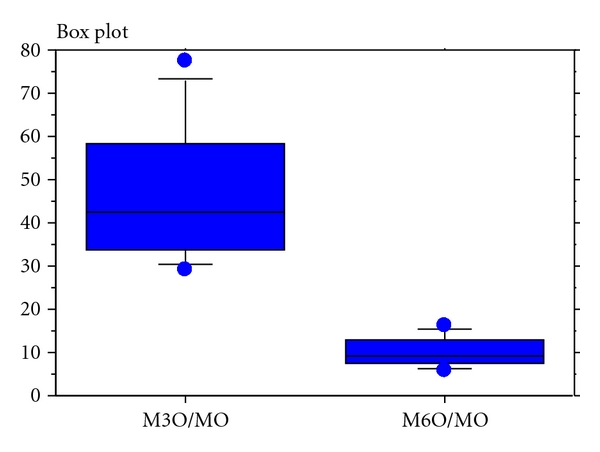
Quotas of oral morphine-3-glucoronide and morphine concentration: M3O/MO and oral morphine-6-glucoronide and morphine concentration: M6O/MO (*n* = 11).

The concentrations of morphine-3-glucoronide (=M3G) and morphine-6-glucoronide (=M6G) were consistently approximately 2.5 times higher after oral administration than after parenteral. The median concentration of M3G divided with M6G was approximately five (4.7–5.0; IQR 0.8–1.1) irrespective of route of administration. Median quota of M3G concentration to morphine concentration was 44 (IQR = 60), and the median quota of M6G:morphine was 10 (IQR = 12) at oral administration (Figure 2).

We measured the symptoms occurring during each administrative method and compared the measurements. We found that the pain levels increased in five patients during parenteral morphine administration (sc *P* = .04, iv *P* = .08). The patients who experienced increased pain during parenteral administration (median 2 steps on the 0–10 NRS scale) did not differ in any significant way regarding patient characteristics (age, gender, diagnosis, ECOG, survival, morphine dose, morphine + M6G concentration, BMI, liver tests, and creatinine) compared with those with stable pain ratings (*n* = 6; Table 1). No increased tiredness could be detected in parenteral administration, but they experienced more nausea during intravenous administration (*P* = .04) compared with oral.

The only significant differences detected between patients experiencing more pain compared with those with maintained pain control were the quotas between oral and subcutaneous M6G concentrations (*P* = .04) and oral and intravenous M6G concentrations (*P* = .01; Table 2). Both M6G oral/sc and M6G oral/iv quotas were significantly lower for those patients experiencing more pain with parenteral administration. It appears that this difference in quotas was mostly due to a higher M6G concentration when taking morphine orally in patients not experiencing increased pain when changing to parenteral administration.

The SD for the log-transformed quota between oral and intravenous concentrations was estimated at 0.38 for the 11 patients. The number of patients needed to reach the power of 60% was 56, and for 80% it was 78 using the equivalence definition 2+/−0.25. The corresponding number using equivalence 2+/−0.2 was 87 and 121, respectively.

## 4. Discussion

This study shows that morphine concentrations are approximately the same—quotas (oral/sc or oral/iv) of 0.88–0.91—if using a conversion factor of 2 to 1, when changing from oral to parenteral administration of morphine. In spite of slightly higher morphine concentrations with parenteral administration, five out of 11 patients experienced more pain. This provokes two thoughts: this suggests that pain might have been even more of a problem if we had chosen a conversion factor of 3 to 1; that is, we had chosen an even lower parenteral morphine dose, and this confirms that morphine concentrations alone cannot be used to predict analgesic effects [9, 10]. 

The interindividual variation of these quotas was rather substantial (from 0.59 to 1.96). This means in practice that, even though the median patient received the same morphine concentrations with this conversion factor, those in the outer ranges might be given anything from double to half the morphine concentration when it is given parenterally compared with when it is given orally.

In this study, no harmful effects correlated to varying morphine concentrations after conversion to parenteral administration could be detected. It still underlines the importance of individual dose titration and the need for an individual evaluation of every patient after a switch has been made from oral to parenteral morphine administration. The identical concentrations of morphine noted in both subcutaneous and intravenous administration support the use of the same conversion factor for both [11]. The finding of identical proportions of metabolite concentrations irrespective of which way of parenteral administration was used further strengthens the same conclusion.

The substantial drop in metabolite concentrations with parenteral administration (median = 2.5 to 1) could be worth remembering in a clinical situation, where side effects because high M3G levels are suspected as a change from oral morphine to parenteral may ease the symptoms. The lower metabolite levels (especially M6G) in parenteral administration may also partly explain the higher pain levels experienced by some patients. As we did not identify any clear-cut differences between those with increased pain and those with continued good pain control except in oral to parenteral M6G quotas, it is perhaps unsafe to draw any firm conclusion beyond saying that this does not contradict the assumption that M6G contributes somewhat to the analgesic effects of morphine [12]. As our study captured merely a week in the lives of our patients whose median survival after study was almost three months, disease progression does not seem to be the most likely explanation of the pain increase. Nevertheless, incurable cancer patients admitted to a palliative homecare team are bound to be suffering from a relatively symptom intensive disease, a situation that requires us to consider the confounding effect that this may have had on pain levels during the study. 

The limited number of participating patients definitely disqualifies us from making any conclusions regarding concentrations or quotas explaining different symptomatic effects. If mass significance can be ruled out, the finding of M6G quotas being involved in pain response may, at the most, be regarded as a finding generating new hypotheses. The calculated quotas of morphine and its metabolites are within the same range as those reported elsewhere [5, 9, 13, 14]. Admittedly, this is not an exact science, especially as the day-to-day variations of registered plasma concentrations in constant doses of oral morphine may vary as much as 18% to 46% [15]. 

This study included cancer patients with continuous morphine therapy. Each studied way of administration was used for at least 48 hours before blood samples were taken. Thus, measured blood concentrations illustrate steady state, as the time limit of five times the halflife of morphine (2 to 4 hours) was well exceeded. Nevertheless, this study does not cover morphine doses higher than low to moderate. Concurrent medications interacting with the metabolism and excretion of morphine may also affect the proposed conversion factor.

To perform an adequately powered study with the same methodology, 121 patients need to be included if we want 80% power and an acceptable range of the conversion factor to be 2+/−0.2. To manage such an effort, a multicentre approach would be the only design with a reasonable chance of success. 

## 5. Conclusion

This pilot study clinically tests the feasibility of a conversion factor of 2 to 1 when changing from oral to parenteral morphine and supports this with concentration measurements of morphine and its metabolites. We did not find any substantial evidence contradicting the advice that a conversion factor of 2 to 1 works rather well in clinical practice. As we found a tendency of less analgesic effect, our conclusion is that the conversion factor definitely should be 2 to 1 as recommended by Takahashi et al. [7] rather than 3 to 1. It is clear that this conversion factor should be used sensitively, and individual monitoring of analgesic effects and side effects are of utmost importance after changing the way morphine is administered. Concurrent medications interacting with morphine metabolism and doses exceeding those examined in this study may necessitate dose titration with some patients. To validate the findings in this pilot study, an adequately powered study would need at least 121 patients.

## Figures and Tables

**Table 1:** Clinical data (*n* = 11).

	Median	IQR	Normal range
Bilirubin	6.0 *μ*mol/L	2.5	3–21
Creatinine	87 *μ*mol/L	50	55–115
ALAT	0.32 *μ*kat/L	0.63	0.00–0.80
ASAT	0.31 *μ*kat/L	0.11	0.00–0.80
BMI*	23	7	
ECOG**	2	1	

*BMI: Body Mass Index.

**ECOG: (1) able to carry out light work, (2) up and about >50% of waking hours, (3) confined to bed or chair >50% of waking hours.

**Table 2:** M6G concentrations and quotas in patients experiencing more pain (yes) in parenteral administration than in oral compared with those with maintained pain control (no).

	Yes (*n* = 5) Median (IQR)	No (*n* = 6)	*P* value median (IQR)
Oral M6G: iv M6G	1.9 (0.28)	2.7 (0.80)	.01
Oral M6G: sc M6G	2.2 (0.85)	3.1 (0.71)	.04

Oral M6G	225 nmol/L (908)	538 (464)	
Iv M6G	120 (544)	168 (186)	
Sc M6G	120 (775)	178 (178)	

## References

[1] WHO (1996). *Cancer Pain Relief: With a Guide to Opioid Availability*.

[2] Hanks GW, Conno FD, Cherny N (2001). Morphine and alternative opioids in cancer pain: the EAPC recommendations. *British Journal of Cancer*.

[3] Gourlay GK (2001). Treatment of cancer pain with transdermal fentanyl. *The Lancet Oncology*.

[4] Jeal W, Benfield P (1997). Transdermal fentanyl: a review of its pharmacological properties and therapeutic efficacy in pain control. *Drugs*.

[5] Glare PA, Walsh TD (1991). Clinical pharmacokinetics of morphine. *Therapeutic Drug Monitoring*.

[6] Lasheen W, Walsh D, Mahmoud F (2010). The intravenous to oral relative milligram potency ratio of morphine during chronic dosing in cancer pain. *Palliative Medicine*.

[7] Takahashi M, Ohara T, Yamanaka H, Shimada A, Nakaho T, Yamamuro M (2003). The oral-to-intravenous equianalgesic ratio of morphine based on plasma concentrations of morphine and metabolites in advanced cancer patients receiving chronic morphine treatment. *Palliative Medicine*.

[8] Zubrod CG, Scheiderman M, Frei E (1960). Cancer—Appraisal of methods for the study
of chemotherapy of cancer in man: thiophosphamide. *Journal of Chronic Disease*.

[9] Mercadant S (1999). The role of morphine glucuronides in cancer pain. *Palliative Medicine*.

[10] Wolff T, Samuelsson H, Hedner T (1995). Morphine and morphine metabolite concentrations in cerebrospinal fluid and plasma in cancer pain patients after slow-release oral morphine administration. *Pain*.

[11] Nelson KA, Glare PA, Walsh D, Groh ES (1997). A prospective, within-patient, crossover study of continuous intravenous and subcutaneous morphine for chronic cancer pain. *Journal of Pain and Symptom Management*.

[12] Faura CC, Moore RA, Horga JF, Hand CW, McQuay HJ (1996). Morphine and morphine-6-glucuronide plasma concentrations and effect in cancer pain. *Journal of Pain and Symptom Management*.

[13] Klepstad P, Kaasa S, Borchgrevink PC (2000). Start of oral morphine to cancer patients: effective serum morphine concentrations and contribution from morphine-6-glucuronide to the analgesia produced by morphine. *European Journal of Clinical Pharmacology*.

[14] Quigley C, Joel S, Patel N, Baksh A, Slevin M (2003). Plasma concentrations of morphine, morphine-6-glucuronide and morphine-3-glucuronide and their relationship with analgesia and side effects in patients with cancer-related pain. *Palliative Medicine*.

[15] Klepstad P, Hilton P, Moen J (2004). Day-to-day variations during clinical drug monitoring of morphine, morphine-3-glucuronide and morphine-6-glucuronide serum concentrations in cancer patients. A prospective observational study. *BMC Clinical Pharmacology*.

